# Improving the Identification of High Risk Precursor B Acute Lymphoblastic Leukemia Patients with Earlier Quantification of Minimal Residual Disease

**DOI:** 10.1371/journal.pone.0076455

**Published:** 2013-10-11

**Authors:** Mawar Karsa, Luciano Dalla Pozza, Nicola C. Venn, Tamara Law, Rachael Shi, Jodie E. Giles, Anita Y. Bahar, Shamira Cross, Daniel Catchpoole, Michelle Haber, Glenn M. Marshall, Murray D. Norris, Rosemary Sutton

**Affiliations:** 1 Children’s Cancer Institute Australia for Medical Research, Lowy Cancer Research Centre, University of NSW, Sydney, Australia; 2 The Oncology Unit, The Children’s Hospital at Westmead, Westmead, Australia; 3 Centre for Children’s Cancer and Blood Disorders, Sydney’s Children’s Hospital, Randwick, Australia; Josep Carreras Leukaemia Research Institute, University of Barcelona, Spain

## Abstract

The stratification of patients with acute lymphoblastic leukemia (ALL) into treatment risk groups based on quantification of minimal residual disease (MRD) after induction therapy is now well accepted but the relapse rate of about 20% in intermediate risk patients remains a challenge. The purpose of this study was to further improve stratification by MRD measurement at an earlier stage. MRD was measured in stored day 15 bone marrow samples for pediatric patients enrolled on ANZCHOG ALL8 using Real-time Quantitative PCR to detect immunoglobulin and T-cell receptor gene rearrangements with the same assays used at day 33 and day 79 in the original MRD stratification. MRD levels in bone marrow at day 15 and 33 were highly predictive of outcome in 223 precursor B-ALL patients (log rank Mantel-Cox tests both P<0.001) and identified patients with poor, intermediate and very good outcomes. The combined use of MRD at day 15 (≥1×10^−2^) and day 33 (≥5×1^−5^) identified a subgroup of medium risk precursor B-ALL patients as poor MRD responders with 5 year relapse-free survival of 55% compared to 84% for other medium risk patients (log rank Mantel-Cox test, P = 0.0005). Risk stratification of precursor B-ALL but not T-ALL could be improved by using MRD measurement at day 15 and day 33 instead of day 33 and day 79 in similar BFM-based protocols for children with this disease.

## Introduction

Acute lymphoblastic leukemia (ALL) accounts for 25% of all cancers in children and cure rates have improved dramatically since the 1970s, so that the 5 year survival rate for paediatric ALL is over 85% in developed countries [1]. Several clinical trials provided strong evidence for the prognostic value of MRD at end of induction and/or consolidation, so that it is now widely used in clinical trials to stratify ALL patients into risk groups [2].

Two recently completed MRD intervention trials, AIEOP-BFM-ALL-2000 and our companion trial ANZCHOG ALL Study 8, stratified patients into the high risk group on the basis of high MRD (>5×10^−4^) at day 79 [3] [4]. This approach was successful in identifying high risk patients but was probably too conservative given the 20% incidence of relapse in the intermediate risk group [5]. Conter et al [5] noted that in their large cohort of 3184 AIEOP-BFM-2000 precursor B-ALL patients 69% (266/387) of the relapses occurred in the intermediate risk group. Their further analysis identified a sub-group of precursor B-ALL patients with a slow early response (SER) to therapy defined as day 33 PCR-MRD≥10^−3^ and day 79 MRD positivity. These SER patients had a 40% cumulative incidence of relapse and are consequently treated more intensively than the other intermediate risk patients in the new AIEOP-BFM-2009 trial [5].

The value of MRD at even earlier timepoints in induction (day 15 or day 19) in the identification of patients with particularly favourable outcomes has already been established for MRD measured by quantitative flow cytometry [6], [7] and in small PCR-MRD studies [8]–[10]. The capacity to also identify high risk patients from day 15 flow-MRD levels has been established for patients enrolled on St Jude Hospital protocols [11] and was suggested by our small PCR-MRD study in ANZCCSG Study 7, a BFM style protocol without MRD intervention [10]. Here we have re-evaluated PCR-MRD at both day 15 and day 33 in ANZCHOG ALL8 patients to determine if earlier time points can identify additional high risk patients in a trial that already used intervention in patients with high MRD at day 79. Since it is now clear that the prognostic significance of MRD at day 33 is different in T-ALL patients [12], we analysed precursor B-ALL patients separately.

## Methods

### Ethical Statement

This study was conducted on 253 patients enrolled at two children’s hospitals between 2002 and 2008 on the ANZCHOG ALL Study 8 clinical trial. This trial, the patient information and consent forms, and MRD analysis were approved by relevant Human Research Ethics Committees (HREC) covering the 3 sites: South Eastern Sydney Area Health Service HREC reference 02/213 for Sydney Children’s Hospital; Children’s Hospital at Westmead Ethics Committee reference HREC 2002/030 and University of New South Wales HREC 06204 to cover MRD analysis at the Children’s Cancer Institute Australia (CCIA). Parental consent was given for all patients at the hospitals and a copy forwarded to CCIA. The release of some samples for this study required approval by the CHW Tumour Bank Committee.

### Clinical Trial Details

The ANZCHOG ALL8 trial was registered on the Australian and New Zealand clinical Trials registry as ACTRN12607000302459 http://www.anzctr.org.au/trial_view.aspx?ID=1568.

### Patients

Patients on this trial were stratified into risk groups using MRD levels at day 33 and day 79 and other risk factors [4]. The high risk group included all patients with at least one high risk feature – poor prednisone response at day 8; not in remission at day 33; MRD>5×10^−4^ at day 79; BCRABL1 positive ALL or positive for the MLL t(4;11) translocation. The standard risk group had no high risk features and were MRD negative at both day 33 and day 79 using two MRD markers with a minimum sensitivity of 10^−4^. The medium risk group were patients not qualifying for either standard or high risk. The stratification was the same as AEIOP-BFM ALL-2000[3] and there were no randomisations. Standard risk and medium risk patients were treated uniformly according to the common control arm in BFM ALL-2000 and high risk patients were assigned to treatment with novel high risk chemotherapy blocks [4].

**Figure 1:**
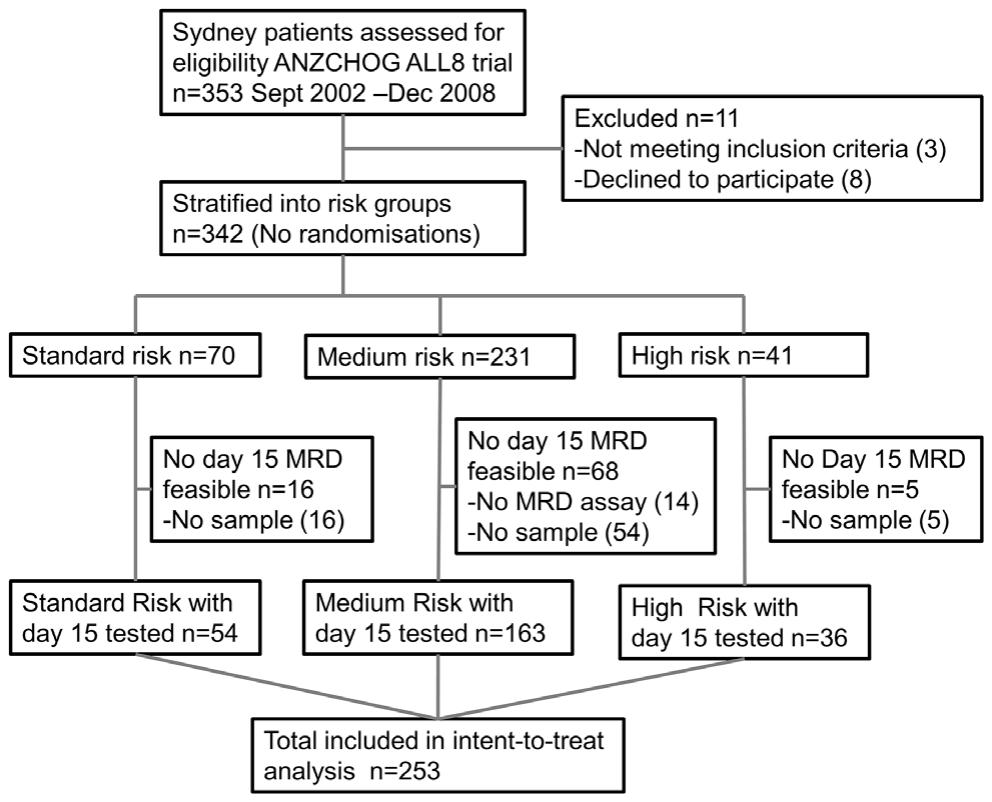
ANZCHOG ALL8 patient samples included in this study. MRD testing and intent-to treat survival analysis was performed on all the feasible patients (n = 253) enrolled between 2002 and 2008 at Sydney Children’s Hospital and The Children’s Hospital Westmead.

Over 600 patients were enrolled in the ANZCHOG ALL8 trial between 2002 and 2011, but this study of day 15 MRD involved only a subset of the 343 patients enrolled at the two Sydney centres in 2002 to 2008 (shown in Figure 1) that included all 253 patients who had stored bone marrow collected at Day 15 and a sensitive MRD assay. In Table 1, the characteristics of the whole group of 343 patients are compared with the 89 patients excluded due to lack of day 15 sample or suitable assay; with the 253 patients included and the 53 included patients who relapsed. There are no substantial differences between the patients analysed for MRD at day 15 and the whole group. The higher proportion of medium risk patients left out of this study reflects the fact that all 14 patients with no suitable MRD assay and no other high risk features were stratified by definition to the medium risk group.

**Table 1:** Characteristics of the patient cohort for this study.

	Sydney cohort		Excluded		Included		Included relapse	
	n = 342		n = 89		n = 253		n = 53	
Standard risk (n)	70	20%	16	18%	54	21%	4	8%
Medium risk (n)	231	68%	68	76%	163	64%	37	70%
High risk (n)	41	12%	5	6%	36	14%	12	23%
Males (n)	200	58%	51	57%	149	59%	37	70%
Females (n)	142	42%	38	43%	104	41%	16	30%
T-ALL (n)	40	12%	10	11%	30	12%	6	11%
prec B-ALL(n)	302	88%	79	89%	223	88%	47	89%
TELAML 1	67	20%	13	15%	54	21%	6	11%
Hyperdiploid>50	64	19%	21	24%	43	17%	7	13%
BCRABL 1	7	2%	0	0%	7	3%	2	4%
B other	164	48%	45	51%	119	47%	32	60%
Median age (yr)	4.8	1.0–17.9	4.0	1.0–17.9	5.0	1.0–17.3	8.2	1.8–17.3
Median WCC	10.3	0.1–547	7.7	0.7–514	11.7	0.1–547	16.4	1.8–17.3

### MRD Analysis

DNA was isolated from either whole bone marrow samples (68% of day 15 samples) or purified mononuclear cells using Nucleobond columns (Machery-Nagel Duren, Germany) and DNA was checked for quality using RQ-PCR for the beta-actin gene as described previously [10]. The rate of MRD positivity at day 15 was the same in DNA from whole bone marrow (93%) and from mononuclear cells (93%). Clonal rearrangements of immunoglobulin and T-cell receptor genes had previously been identified for each patient by PCR and sequencing. With reference to the NCBI IgBlast (www.ncbi.nlm.nih.gov/igblast/) database, real-time quantitative PCR assays were designed for at least one marker and preferably two markers for each patient based on patient specific primers and consensus probes [10], [13]. The MRD tests were performed on a Biorad IQ-5 platform with standards made by serial dilution of the patient’s diagnostic DNA (1×10^−1^, 1×10^−2^, 1×10^−3^, 5×10^−4^, 1×10^−4^, 5×10^−5^ and 1×10^−5^) and were analysed according to EuroMRD guidelines [14]. The day 15 MRD analysis was performed retrospectively using the more sensitive or the first marker used for stratification.

### Statistics

Relapse-free survival (RFS) was defined as time from remission to either relapse or last clinical follow up. Four patients were censored at the time of diagnosis of a second malignancy and 4 patients were censored at time of death either in induction (n = 1) or in remission later in therapy (n = 3). Receiver operating characteristic (ROC) analysis was performed using Medcalc to estimate the best discriminatory thresholds for MRD [15]. Kaplan Meier Survival curves and Cox-Mantel log rank analysis was performed using Graphic Pad Prism and Medcalc was used for the Cox proportional hazard model of multivariate analysis.

## Results

**Figure 2:**
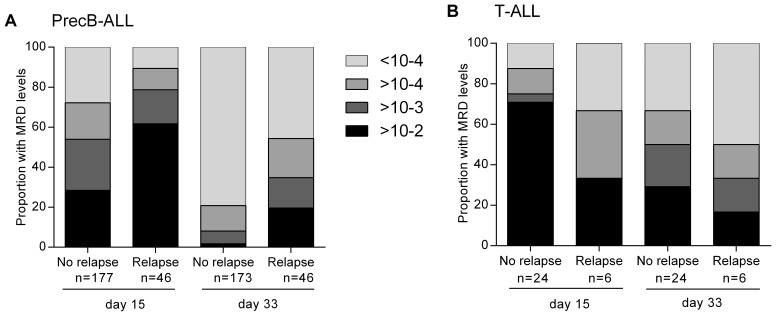
Evaluation of MRD and relapse in paediatric ALL patients. The proportion of patients with low MRD (<10^−4^), moderate (≥10^−4^, <10^−3^), high (≥10^−3^<10^−2^) and very high MRD (≥10^−2^) at day 15 and day 33 by relapse status for A) precursor B and B) T-ALL patients.

The MRD results at day 15 and day 33 were first evaluated by comparing the proportion of patients with low, moderate, high and very high levels of MRD in relapsed and non-relapsed patients (Figure 2). In precursor B-ALL, patients who later relapsed were more likely to have higher levels of MRD at day 15 and at day 33 (Figure 2A). The median level of MRD at day 15 was 2×10^−2^ for relapsed patients versus 1×10^−3^ in patients still in remission and decreased to 2×10^−4^ at day 33 for relapsed and non-quantitative positivity (<1×10^−4^) for non-relapsed patients. The same effect was not seen in T-ALL patients (Figure 2B). Overall, MRD levels were also usually higher in T-ALL patients (with a median of 2×10^−2^ compared to 4×10^−3^ in precursor B-ALL at day 15). The low number of relapses (6/30) precluded further analysis but our results for T-ALL were consistent with a slower MRD response to therapy in T-ALL and with the AIOEP-BFM report that the 32% of T-ALL patients who are MRD positive at day 33 and achieve MRD negativity at day 79 still have an excellent prognosis [5].

**Figure 3:**
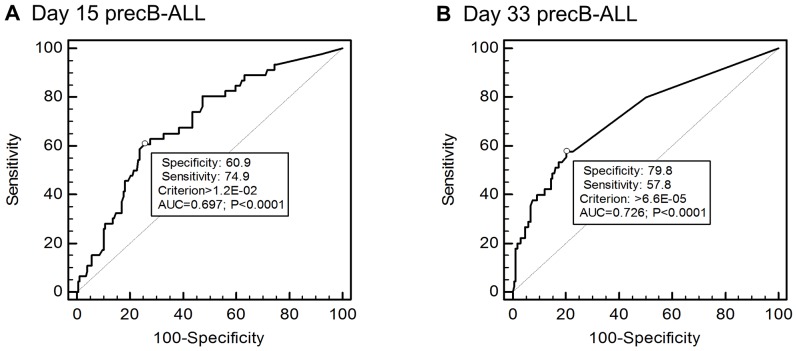
Assessment of optimal thresholds for MRD prediction of relapse. Receiver Operating Characteristic (ROC) curves show the relationship between true-positive (Sensitivity) and false-positive (100-Specificity) rates at different thresholds with the optimal MRD threshold marked with a ° symbol on the curve. AUC = Area under the curve and the P value is the probability that AUC = 0.5 (shown by the dotted line) which represents a test with no predictive value. ROC curves are shown for (A) Day 15 MRD to predict relapse in 223 precursor B-ALL ANZCHOG ALL8 patient and (B) Day 33 MRD in 219 of the 223 patients (4 patients lack sufficient DNA for analysis). ROC curves for T-ALL patients at both timepoints did not reach significance.

The optimal MRD thresholds at day 15 and day 33 to partition patients were assessed using Receiver operating characteristic (ROC) curves (Figure 3A, B). ROC analysis is used to assess the diagnostic accuracy of a continuous variable and to estimate the threshold which optimizes the balance between sensitivity (true positive rate) and specificity (true negative rate) [15], [16]. The ROC analysis of area under the curve was not statistically significant for relapse events and MRD at day 15 or day 33 MRD in T-ALL patients. In contrast it was significant for precursor B-ALL patients at both time points. The most accurate threshold to detect relapse in the 223 precursor B-ALL patients was estimated as MRD 1.2×10^−2^ at Day 15 and 6.6×10^−5^ at Day 33. These thresholds, rounded to the nearest half log value, were applied in survival analyses.

**Figure 4:**
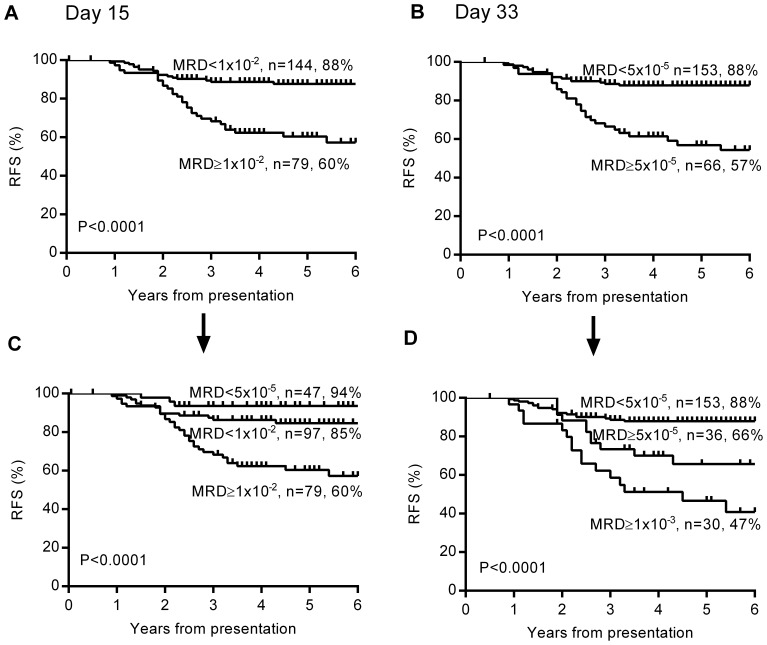
Prognostic value of MRD at day 15 and day 33 in precursor B-ALL. Kaplan Meier survival curves based on the MRD thresholds determined by ROC analysis are shown for A) 223 precursor B-ALL patients split according to Day 15 MRD B) the same patients based on Day 33 MRD. A second MRD threshold is added to identify 3 risk levels for both C) Day 15 MRD and D) Day 33 MRD. The number of patients and 5 year relapse-free survival percentage are given for each subgroup.

The resulting Kaplan Meier analysis showed that MRD at day 15 (≥1×10^−2^) was highly predictive of patient outcome in the precursor B-ALL cohort with RFS of 60% compared to 88% (log rank Mantel-Cox test P<0.0001, Figure 4A). Similarly, day 33 MRD≥5×10^−5^ defined a group with 57% RFS versus 88% (P<0.0001, Figure 4B). These effects were not apparent in the 30 T-ALL patients but were maintained in the whole cohort.

Previous studies and the patient characteristics shown in Table 1, suggested that other factors may influence relapse outcomes. A multivariate Cox proportional hazard model of RFS in precursor B patients was fitted for 7 prognostic factors – gender, age, white cell count (>50,000/ul),and favourable cytogenetics (hyperdiploidy>50 or TELAML1 positivity) at diagnosis and MRD at day 15, day 33, and day 79. The three covariants retained in the model were MRD at day 15 and day 33 and age ≥10 years with relative hazards and 95% confidence intervals of 2.3 (1.1–4.6); 1.9 (0.97–3.9) and 2.2 (1.1–4.0) respectively. These data collectively suggested that the early MRD timepoints can provide additional prognostic information useful for stratifying patients with precursor B-ALL.

From a clinical perspective, ALL patients have been stratified into high, intermediate and standard treatment risk groups. We therefore arbitrarily defined an extra MRD threshold for precursor B-ALL patients at both day 15 and day 33 in order to distinguish 3 risk groups with a reasonable number of patients (Figure 4C, 4D). At day 15, the use of a lower threshold (<5×10^−5^), which includes patients with both no detectable MRD and those positive below the quantifiable range of the MRD assay, defined a group with very favourable outcomes (n = 47, 94% RFS) consistent with previous findings [8]–[10] (Figure 4C). At day 33, PCR-MRD≥1×10^−3^ was chosen as the additional cutpoint based on results of the AIEOP-BFM 2000 trial [5] and earlier studies including our own [17]. Patients with day 33 MRD≥1×10^−3^ (n = 30) had an RFS of 47% defining the highest risk group (Figure 4D).

An analysis of the incidence of 46 relapses occurring in precursor B-ALL patients showed that 33 (72%) occurred in the medium risk group of 150 patients. Reclassification by the new MRD criteria adopted by the AIEOP-BFM-ALL 2009 trial would move 12 of these patients with 5 relapses (42%) into the slow early responder group (defined as precursor B-ALL with MRD>5×10^−4^ at day 33 and MRD positive<5×10^−4^ at day 79). While this confirms the finding that these patients are at a higher risk, it also shows that this new stratification system still missed a significant number of relapses in our cohort (n = 28). Our results suggested that use of MRD criteria based on a single timepoint (day 15 or day 33) would not be helpful but that stratification could be improved by using both early timepoints.

**Figure 5:**
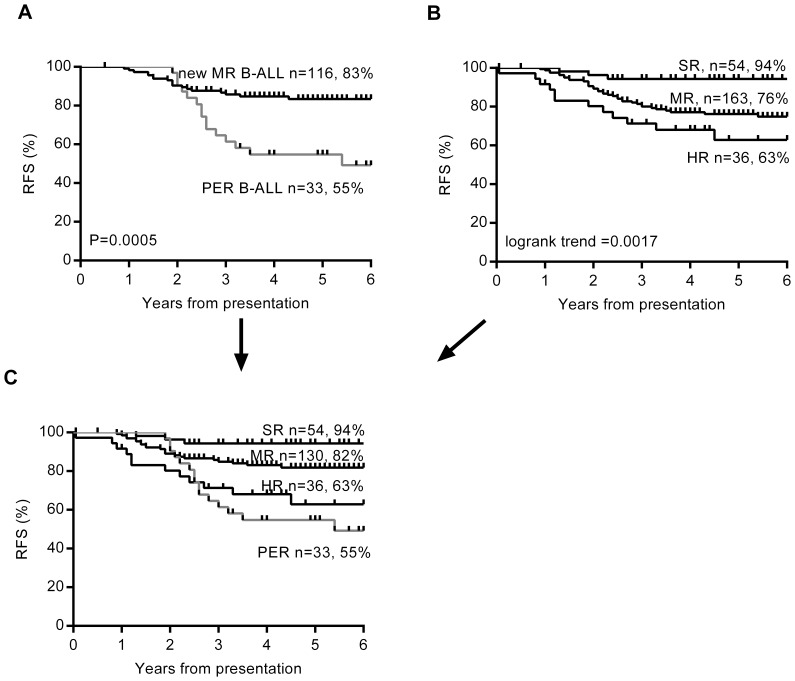
Identification of a new poor early response group within medium risk for MRD risk stratification. Kaplan Meier survival curves are shown for A) the 150 medium risk precursor B-ALL patients split into poor early responders (PER) defined as MRD day 15≥1×10^−2^ AND day 33 MRD≥5×10^−5^ and good molecular responders with MRD day 15<1×10^−2^ OR day 33 MRD<5×10^−5^ B) The original stratification of whole cohort of 253 patients into standard risk (SR), medium risk patients (MR) and high risk (HR) groups according to ANZCHOG ALL8 protocol criteria and C) the whole cohort stratified according to the new criteria with the PER subset of medium risk B-ALL patients defined separately. The number of patients and 5 year relapse-free survival percentage are given for each subgroup.

Our analysis showed that more intermediate risk patients with poor outcome can be identified using day 15 MRD≥1×10^−2^ in combination with day 33 MRD≥5×10^−5^ (Figure 5A). The combined poor MRD response at day 15 and day 33 identified 33 medium risk patients as higher risk, 14 (44%) of whom relapsed. We have called this subset of medium risk B-ALL patients “poor early responders” (PER), to distinguish it from the SER risk category used in our current AIEIOP-BFM trial. The potential benefit of this alternative patient stratification is clear from a comparison of the original stratification (Figure 5B) and the new alternative (Figure 5C).

With the new stratification (Figure 5C), the 5 year RFS in precursor B patients was substantially lower for PER patients in the medium risk group (55%) compared to good responders in medium risk (82%) and standard risk patients (94%), and lower than high risk patients (63%) treated more intensively. It is also important to note that these criteria identified 13 of the 14 precursor B-ALL patients originally stratified as high risk due to high MRD at day 79. The exception was a patient with very unusual MRD kinetics with MRD increasing by more than one log level at both day 33 and day 79.

## Discussion

This study has shown in children treated for acute lymphoblastic leukaemia that the rapid clearance of bone marrow disease is associated with a low relapse rate and conversely that patients with high levels of disease have higher rates of relapse.

It is not surprising that MRD measured by PCR at day 15 is predictive of poor as well as excellent outcomes in patients treated on BFM protocols. The morphological examination of bone marrow aspirates at day 15 was established early as a prognostic indicator of poor outcome [18] and this remains relevant in BFM trials particularly when MRD measurement is not feasible [19]. Flow-MRD at earlier time points for patient stratification has already been used in the St Judes Total XV and XVI protocols (after 2 weeks of induction) and COG protocols (day 29) [11]. European trials using PCR-MRD at day 28 or 29 include the UKALL 2003 trial and NOPHO ALL-2008 [2]. The findings of Basso et al [6] led to the use in the current AIEOP-BFM trial of day 15 flow to identify low risk patients by flow-MRD and slow early responders at day 33 although there is still a requirement for day 79 MRD>5×10^−4^ or positivity for stratification into high or slow early responder group respectively [5].

The separate analysis of precursor B and T-ALL patients in BFM protocols has improved our understanding of response to therapy and risk [5], [12], [15], [19]. In patients receiving current AIEOP-BFM therapy, MRD at day 79 remains critically important in T-ALL whereas early timepoints are needed to maximise the numbers of high risk patients identified. MRD over-rides the significance of poor prednisone response in precursor B-ALL but not T-ALL [19]. Earlier stratification of high risk patients in clinical trials may be beneficial in enabling novel treatments to be trialled on patients who achieve only a shallow remission at the end of induction with reductions in MRD providing a surrogate end-point. PCR-MRD methodologies are now well established and the development of PCR-MRD assays for each patient by day 33, involving target detection, sequencing and primer design, is no longer a difficult challenge. While there is good reason to delay stratification for T-ALL patients in whom the day 79 MRD results provide better prognostic discrimination [12] our analyses suggest that risk assessment of precursor B-ALL can be improved by the combined use of day 15 and day 33 MRD results to identify the PER group.

Further study of the value of day 15 MRD is needed to overcome the limitations inherent in doing a retrospective study on an incomplete set of patient samples. A larger cohort would enable a better analysis of all factors contributing to patient outcomes, The prospective analysis and comparison of MRD measured by PCR and quantitative flow cytometry in the ongoing AIEOP-BFM ALL 2009 trial that will accrue 5000 patients will be useful to confirm these findings and to extend our understanding of different ALL subtypes.

It is clear that the use of MRD (>5×10^−4^) at day 79 to identify high risk patients in ANZCHOG ALL8 was conservative and predicted fewer relapses than is possible from MRD at earlier time points. MRD at day 15 of therapy provided additional predictive value in precursor B-ALL patients treated on this MRD intervention protocol and could be used in future to identify additional patients at high risk of relapse. Better tailoring of treatments to suit different subsets of ALL patients could lead to further improvements in morbidity and mortality for ALL patients.
